# Transcatheter Aortic Valve Replacement: Latest Advances and Prospects

**DOI:** 10.3390/jcm13216583

**Published:** 2024-11-01

**Authors:** Lluis Asmarats, Dabit Arzamendi

**Affiliations:** 1 Cardiology Department, Hospital Santa Creu i Sant Pau, Biomedical Research Institute (IIB Sant Pau), 08025 Barcelona, Spain; 2 Centro de Investigación Biomédica en Red de Enfermedades Cardiovasculares (CIBERCV), 28029 Madrid, Spain

## 1. Introduction

Aortic stenosis is the most common degenerative valvular heart disease leading to surgery or transcatheter therapy in Europe and North America. Over the past two decades, transcatheter aortic valve replacement (TAVR) has revolutionized the management of symptomatic severe aortic stenosis, and has become the treatment of choice for patients aged ≥75 years across the surgical risk spectrum [1]. Since the first-in-man case in 2002 [2], continuous device iterations, increased operator experience, and procedure simplification have led to a dramatic reduction in procedural complications and steady improvement in clinical outcomes [3]. However, despite these improvements, complications still occur in a non-negligible proportion of patients in contemporary practice and may adversely affect clinical outcomes. Therefore, continuous efforts seek to minimize some of the knowledge gaps and unresolved issues, with a particular focus on younger low-risk patients with longer life expectancies. Relevant topics of concern include new-onset conduction disturbances, bicuspid aortic valve, and the feasibility of redoTAVR and coronary access. This Special Issue aims at addressing some of these gaps, from pre-procedural planning to post-procedural management, to further improve clinical outcomes and ensure the best lifetime management for patients.

## 2. Conduction Disturbances

New-onset conduction disturbances remain the most common complication after TAVR, given the proximity between the aortic valve and the conduction system, and constitutes the Achilles heel of this therapy and probably the main limitation compared with surgery. The incidence of permanent pacemaker (PPM) after TAVR ranged from 2% to 51%, with a pooled rate of 13%, in an analysis including more than 16,000 patients [4,5]. Whereas several predictors of PPM post-TAVR have been reported (first-degree atrioventricular block, implantation depth, and self-expanding valves), baseline right bundle branch block (RBBB) is likely the strongest predictor of PPM [4]. In this Special Issue of the *Journal of Clinical Medicine*, Schoechlin et al. (Contribution 1) analyzed the risk factors for PPM post-TAVR among patients with preexisting RBBB. These patients exhibited a high risk of PPM (~40%), especially women, patients receiving a self-expanding valve, and those with greater calcification of the non-coronary left ventricular outflow tract. These findings highlight the importance of close electrocardiographic monitoring and surveillance in this high-risk subset of patients.

## 3. Antithrombotic Therapy Post-TAVR

The optimal postprocedural antithrombotic therapy after TAVR has been controversial during the past decade. Although dual antiplatelet therapy was recommended in the early era of TAVR for patients without an indication for chronic anticoagulation, several randomized trials (Aspirin Versus Aspirin + Clopidogrel Following Transcatheter Aortic Valve Implantation [ARTE] and AntiPlatelet therapy fOr Patients Undergoing Transcatheter Aortic Valve Implantation [POPular-TAVI]) suggested that single antiplatelet therapy was associated with a significant reduction in bleeding complications without a significant increase in ischemic events [6,7]. Accordingly, the 2021 European guidelines granted a class I-A recommendation for lifelong single antiplatelet therapy post-TAVR in patients with no indication for long-term anticoagulation [1]. In a meta-analysis including close to 3000 patients, Abuelazm et al. (Contribution 2) evaluated the safety and efficacy of direct oral anticoagulants (DOACs) versus the standard of care in TAVR candidates without an indication of anticoagulation. DOACs reduced the risk of hypoattenuated leaflet thickening, but were linked to increased all-cause mortality (mainly non-cardiovascular), therefore supporting the general recommendation of a more conservative antithrombotic approach in current guidelines.

## 4. Lifetime Management of Aortic Stenosis

As TAVR expands into younger, low-risk patients, several concerns of major clinical importance have emerged, such as the management of patients with bicuspid aortic valve (BAV), transcatheter heart valve (THV) durability, coronary access post-TAVR, and the need for reintervention after TAVR.

BAV disease is the most common congenital cardiac disease with a reported prevalence greater than 60% in patients under 70 years [8]. In a recent study by Gupta et al. [9], more than half of patients under 65 years with severe aortic stenosis received TAVR in the United States, suggesting an increase in the number of patients with BAV undergoing TAVR in the coming years. Patients with BAV were excluded from the landmark randomized trials, and albeit several registries showed similar results between bicuspid and tricuspid valves with latest-generation THV devices, randomized clinical trials comparing TAVR and surgical aortic valve replacement in patients with BAV are lacking [10]. In the present Special Issue, Gutiérrez et al. (Contribution 3) provide an overview of TAVR in BAV patients, from pre-procedural planning to clinical outcomes. The authors describe different sizing methods for BAV (annular vs. supra-annular, circle method, LIRA, etc.) and provide a step-by-step guide for procedural implant and the positioning of different transcatheter prostheses to avoid complications and optimize clinical outcomes in this challenging scenario.

The expansion of TAVR to younger patients has raised some concerns regarding THV durability and the need for future reinterventions. Whereas a growing body of evidence supporting the use of TAVR in degenerated surgical aortic bioprosthesis exists [11], pre-procedural planning of redo TAVR remains complex and data on the use of TAVR within failed THV are scarce [12]. In this Special Issue, Galhardo et al. (Contribution 4) discuss which patients with degenerated THV may benefit from either redo TAVR or TAVR explantation, and provide essential concepts to select the appropriate candidates for redo TAVR, as well as critical aspects of pre-procedural computed tomography planning and how to prevent procedural complications inherent to this technically demanding procedure.

Commissural alignment has gained increasing relevance in the lifetime management of patients with aortic stenosis, in order to facilitate future coronary re-access (particularly in young patients with longer life expectancy), to enable the use of leaflet modification techniques in patients at risk for coronary obstruction, and to optimize THV performance and durability [13]. Paredes-Vazquez et al. (Contribution 5) provide an in-depth overview of the incidence and clinical impact of commissural misalignment in TAVR, and offer a detailed description of how to achieve optimal commissural and coronary alignment with different THV platforms.

## 5. Ongoing Trials and Future Directions

The excellent outcomes achieved with TAVR with newest-generation devices will likely fuel an expansion of the therapy toward novel clinical indications. The optimal timing of aortic valve replacement in patients with severe asymptomatic aortic stenosis has been questioned. Two randomized trials (Randomized Comparison of Early Surgery versus Conventional Treatment in Very Severe Aortic Stenosis [RECOVERY] and Aortic Valve Replacement Versus Conservative Treatment in Asymptomatic Severe Aortic Stenosis [AVATAR]) previously showed a reduction in all-cause mortality with a strategy of early surgical replacement [14,15]. Several trials are currently evaluating the potential role of early TAVR in patients with asymptomatic severe aortic stenosis (Early valve replacement in severe ASYmptomatic Aortic Stenosis [EASY-AS, NCT04204915], and Evaluation of TAVR Compared to surveillance for patients with Asymptomatic Severe Aortic Stenosis [EARLY-TAVR, NCT03042104]) or with moderate aortic stenosis (Transcatheter Aortic Valve Replacement to Unload the Left Ventricle in Patients With ADvanced Heart Failure [TAVR-UNLOAD, NCT02661451], Prospective Randomized Controlled Trial to Assess the Management of Moderate Aortic Stenosis by Clinical Surveillance or Transcatheter Aortic Valve Replacement [PROGRESS, NCT04889872], and Evolut EXPAND TAVR II Pivotal Trial [NCT05149755]) [16,17]. Moreover, whereas several observational studies have suggested TAVR to be a reasonable option for patients with aortic stenosis and BAV, randomized clinical trials comparing surgical aortic valve replacement to TAVR in BAV patients are eagerly awaited. Finally, evidence on TAVR for treating pure native aortic regurgitation is still scarce, and additional studies evaluating the safety, efficacy, and long-term performance of TAVR in patients with pure aortic regurgitation using new dedicated THV devices are needed.
